# Multi-view knowledge-guided flow subgraphs with substructure initialization for explainable DDI prediction

**DOI:** 10.1093/bfgp/elag006

**Published:** 2026-07-01

**Authors:** Yugui Xu, Zhigan Zhou, Hao Yuan, Siqi Liu, Quan Zou, Yongqing Zhang, Wenqian Zhang

**Affiliations:** School of Computer Science, Chengdu University of Information Technology, No. 24 Block 1, Xuefu Road, Chengdu, 610225, China; School of Computer Science, Chengdu University of Information Technology, No. 24 Block 1, Xuefu Road, Chengdu, 610225, China; School of Computer Science, Chengdu University of Information Technology, No. 24 Block 1, Xuefu Road, Chengdu, 610225, China; School of Computer Science, Chengdu University of Information Technology, No. 24 Block 1, Xuefu Road, Chengdu, 610225, China; Institute of Fundamental and Frontier Sciences, University of Electronic Science and Technology of China, No. 2006, Xiyuan Ave, West Hi-Tech Zone, Chengdu 611731, China; School of Computer Science, Chengdu University of Information Technology, No. 24 Block 1, Xuefu Road, Chengdu, 610225, China; Department of Thoracic Surgery, Beijing Chaoyang Hospital, Capital Medical University, XiYuan Campus, 5 Jingyuan Road, Shijingshan District, Beijing, 100043, China

**Keywords:** drug–drug interaction, multi-view knowledge, graph neural networks, contrastive learning

## Abstract

Polypharmacy has become essential in managing complex and chronic conditions, yet it introduces significant clinical risks due to potential drug–drug interactions (DDIs). Existing computational models struggle to provide accurate and interpretable predictions, largely due to fragmented integration of molecular structures and biomedical knowledge. These limitations arise from static graph designs, inadequate substructure modeling, and insufficient incorporation of semantic context, highlighting the need for a unified, explainable solution. In this study, we propose MKGFlow-DDI, a multi-view knowledge-guided framework that jointly leverages drug–drug interaction networks and biomedical knowledge graphs to dynamically construct drug-flow subgraphs. The model incorporates a dual-channel encoder designed to capture atom-level information and substructure-level features, integrating them with the global semantic embeddings of the composite network to derive novel feature representations. These representations are fused to initialize node features within each subgraph, which are iteratively optimized through similarity-based edge refinement to reduce noise and enhance biological relevance. To further improve generalization and stability, a contrastive learning module is introduced to align representations of perturbed subgraphs by maximizing consistency across positive and negative sample pairs. Experimental results on DrugBank and TWOSIDES demonstrate that MKGFlow-DDI outperforms state-of-the-art baselines, especially in scenarios involving previously unseen drugs. Additionally, the model produces interpretable semantic pathways that align with known pharmacological mechanisms, enabling clinically meaningful insights. Overall, MKGFlow-DDI establishes a robust and biologically grounded approach to DDI prediction, offering a promising direction for computational pharmacovigilance and personalized therapy optimization.

## Introduction

Clinical manifestations in patients with chronic diseases, infections, or aging frequently arise from complex, multifactorial pathophysiological mechanisms [1, 2]. Under these conditions, monotherapy often fails to achieve optimal therapeutic outcomes [3]. Combination therapy, conversely, offers distinct clinical advantages, including enhanced efficacy, reduced resistance development, and improved management of recurrent conditions with multiple comorbidities [4]. However, polypharmacy introduces significant safety concerns, primarily elevated risks of unexpected drug–drug interactions (DDIs) [5]. These interactions originate from diverse biological mechanisms, including metabolic enzyme induction, competition at shared receptor sites, and interference with downstream signaling cascades. They can alter pharmacokinetic and pharmacodynamic profiles and, in severe cases, precipitate adverse clinical outcomes. The inherent complexity of these mechanisms, compounded by pharmaceutical proliferation and combinatorial expansion of therapeutic regimens, underscores the urgent need for robust DDI prediction strategies [6].

Traditional methodologies, including *in vitro* assays and clinical trials, retain value but face mounting limitations: prohibitive costs, extended timelines, and inadequate scalability to accommodate continuous drug development [7]. Furthermore, newly approved drugs often lack comprehensive post-marketing surveillance data, complicating interaction profile assessment. This critical gap necessitates efficient, scalable, and interpretable computational models to enable early-stage pharmacovigilance and personalized therapeutic decision-making. Early computational approaches for DDI prediction predominantly employed traditional machine learning (ML) techniques based on the drug similarity hypothesis, which posits that drugs with similar structures, functions, or phenotypes tend to exhibit correlated interaction profiles. Representative models, including INDI [8] and PEA [9], constructed drug-pair features using handcrafted similarity metrics derived from chemical structures, side-effect profiles, gene expression, or metabolic pathways. While providing foundational insights with moderate predictive performance, these approaches suffer critical limitations: dependence on manually curated features and domain knowledge constrains scalability and generalizability, while performance degrades in high-dimensional, noisy, or sparse data environments. To address these constraints, deep learning (DL) has emerged as a promising alternative, capable of autonomously learning complex nonlinear representations from heterogeneous inputs [10]. For instance, DeepDDI [11] established an end-to-end framework predicting 86 DDI types exclusively from drug structures, while GMPNN-CS [12] leveraged graph message-passing networks to extract fine-grained molecular substructures, generating interpretable attention maps. Recent fusion architectures such as MDF-SA-DDI [13] and SRR-DDI [14] incorporated attention mechanisms and substructure refinement strategies to better capture synergistic drug-drug effects. Despite substantial progress in representation learning, most DL models remain largely data-driven and fail to adequately capture the semantic, relational, and mechanistic foundations of DDIs. More critically, their limited interpretability, which often results in predictions lacking biologically traceable rationales, substantially constrains their practical value in clinical decision support and pharmacovigilance.

To address contextual and mechanistic information deficits in DL models [15], researchers increasingly leverage biomedical knowledge graphs (KGs). These structured repositories encode rich multi-relational information among drugs, proteins, pathways, and diseases, enabling semantic reasoning and relation-aware learning. Models like Conv-LSTM [16] integrated graph-based embeddings from DrugBank, KEGG, and PharmGKB to incorporate domain knowledge. SumGNN [17] employed subgraph-level self-attention for context-specific semantics, while LaGAT [18] introduced link-aware graph attention to dynamically select paths based on drug-pair dependencies. KGCNN [19] employs spatial-aware capsule aggregators within Graph Neural Networks (GNNs) to capture molecular relationships for DDI prediction. Know-DDI [20] achieved interpretable DDI prediction through knowledge subgraph learning. Recent large language models (LLMs) facilitate hybrid frameworks that fuse structured KGs with unstructured biomedical corpora [21, 22]. LLM-DDI employs message-passing GNNs enhanced by GPT embeddings to model biomedical KGs. Despite progress, current KG-augmented models face persistent limitations: isolated processing of drug structures and semantic contexts, inadequate mechanisms for dynamically prioritizing relevant subgraphs, rare generation of biologically aligned interpretable paths, and restricted generalization to novel drugs due to reliance on static subgraphs or precomputed embeddings.

To overcome these challenges, we propose MKGFlow-DDI—a multi-view Knowledge-Guided Subgraph Flow framework with Substructure Initialization—designed to enhance the accuracy, robustness, and interpretability of DDI prediction. First, we construct a composite relational graph by integrating external biomedical KGs with empirical DDI networks, enabling dynamic extraction of context-aware flow subgraphs that capture structural dependencies and semantic interactions. Second, a dual-channel encoder performs hierarchical representation learning: GINEConv extracts atom-level chemical features while SAGPool derives substructure-level representations via hierarchical pooling, fused with global GraphSAGE embeddings for multi-scale (MS) knowledge-enriched nodes. Third, semantic edge filtering adaptively emphasizes high-similarity relations while pruning irrelevant connections, enhancing subgraph coherence and reducing noise. Fourth, contrastive subgraph learning is constructed positive/negative subgraph pairs to enhance inter-class separability and enforce cross-modal consistency, improving generalization in data-scarce. Finally, the framework supports traceable semantic path reasoning, providing biologically grounded explanations through clinically meaningful relational pathways for real-world deployment.

We extensively evaluate MKGFlow-DDI on benchmark datasets: DrugBank and TWOSIDES, addressing challenges including class imbalance, semantic noise, and heterogeneous data integration. The framework consistently outperforms state-of-the-art baselines across metrics (AUC, F1-score), demonstrating superior robustness and generalization capability. Qualitative case studies further reveal interpretable pathways aligned with pharmacological mechanisms, highlighting its potential in pharmacovigilance and therapeutic design.

## Related work

This chapter reviews recent advances in DDI prediction, encompassing traditional machine learning methods, custom strategy-based approaches, DL techniques, and emerging methodologies that integrate KGs. It also examines pertinent work on graph neural networks (GNNs) and contrastive learning, establishing the theoretical and technical foundation for subsequent model design.

### Drug–drug interaction event prediction

Existing methodologies for predicting DDI events are broadly categorized into machine learning-based, non-machine learning-based, and DL-based approaches. Cheng *et al.* [23] proposed HNAI, which analyzes similarity across four drug feature types and integrates five machine learning models to infer DDIs under diverse similarity scenarios. Non-traditional machine learning methods typically employ customized strategies or specific assumptions during model construction. For instance, Zhang *et al.* [24] developed an integrated label propagation framework leveraging multi-source data for DDI event prediction. The rapid advancement of DL has spurred numerous DDI prediction methods utilizing graph embedding techniques such as DeepWalk [25] and node2vec [26], which learn drug similarities and relational patterns to achieve promising predictive performance. More recently, contrastive learning, originally developed in computer vision, has been adapted to DDI prediction [27]. By constructing positive and negative sample pairs, this approach facilitates learning of effective representation spaces to enhance prediction accuracy. Xiong *et al.* [28] exemplified this through a multi-relational contrastive learning GNN featuring a dual-view negative adversarial augmentation strategy, enabling multi-relation contrastive learning that effectively captures latent information in rare DDI events [29–31].

### GNN-based methods

GNN-based DDI prediction approaches generally operate at either atom-level or molecule-level representations. Atom-level methods model atoms as nodes and chemical bonds as edges. Zhong *et al.* [32] developed DDI-graph convolutional network (GCN), employing GCNs [33] to aggregate neighborhood information and generate graph-level features for effective DDI prediction. SSI-DDI [34] incorporates graph attention networks (GAT) [35] to operate directly on original molecular graph representations, enabling richer feature extraction while reformulating DDI prediction as modeling pairwise interactions between fundamental drug substructures. Molecule-level GNNs represent entire drug molecules as nodes, with interaction types between drug pairs constituting edges. Zitnik *et al.* [36] developed a multi-relational link prediction method where nodes connect via multiple edge types within multi-view graphs, enabling prediction of specific adverse effects for clinical drug combinations. SkipGNN [37] introduced MS skip-feature aggregation through integration of original graphs with skip graphs constructed using second-order similarity, thereby enhancing GNN performance [38–40].

### Knowledge graph-based methods

KG applications in DDI prediction have achieved significant progress, primarily through structured enrichment of drug representations [41]. Lin *et al.* [42] developed the end-to-end knowledge graph neural network (KGNN) framework, which captures drug-neighbor associations by mining relational paths within KGs. To enhance modeling of high-order structural and semantic relationships, KGNN learns topological information for each entity while expanding its receptive field. Su *et al.* [43] incorporated attention mechanisms into the KG-based (DDKG) framework, initializing drug representations via attribute embeddings and recursively aggregating neighborhood information along paths to fully leverage drug attributes and triple-based factual data. Zhang *et al.* [44, 45] proposed learning-based DDI prediction models employing incremental sampling mechanisms, while Ren *et al.* [45] designed a biomedical KG framework incorporating local and global information. SumGNN [17] utilizes self-attention mechanisms to summarize subgraphs and generate biologically meaningful reasoning paths, constructing sparse yet efficient subgraph representations. In contrast, MKGFlow-DDI dynamically constructs pair-specific drug-flow subgraphs and integrates dual-channel feature fusion with iterative edge refinement. This design enables context-aware interaction modeling and progressive noise suppression beyond predefined graph structures.

## Materials and methods

This section systematically introduces the comprehensive design framework of MKGFlow-DDI, encompassing key technical components such as problem definition, combinatorial graph construction, drug substructure feature extraction, subgraph initialization and refinement mechanisms, and contrastive learning strategies. This structured approach provides a solid foundation for the model’s predictive performance and interpretability.

### Problem definition

In this section, we formally define the DDI prediction task addressed in this study. The DDI graph is denoted as $\mathcal{N}_{\mathtt{D}} = \{\mathcal{V}_{\mathcal{D}}, \mathcal{E}_{\mathcal{D}}, \mathcal{R}_{\mathcal{D}}\}$, which encodes interaction information among drugs. Here, $\mathcal{V}_{D}$ represents the set of drug nodes, $\mathcal{E}_{D}$ denotes the set of edges, and $\mathcal{R}_{D}$ refers to the set of relation types. Specifically, each edge in the DDI graph corresponds to a triple $(u, r, v)$, where $u, v \in \mathcal{V}_{D}$ and $r \in \mathcal{R}_{D}$. The external KG is denoted as $\mathcal{N}_{K} = \left \{ \mathcal{V}_{K}, \mathcal{E}_{K}, \mathcal{R}_{K} \right \}$, which contains rich biomedical information among various entities. In this representation, $\mathcal{V}_{K}$ includes head and tail entities, $\mathcal{R}_{K}$ represents the interaction types between entities (e.g. between drugs and genes), and each edge $(u, r, v) \in \mathcal{E}_{K}$ denotes a factual triple in the KG. The DDI graph $\mathcal{N}_{D}$ and the external KG $\mathcal{N}_{K}$ are then integrated into a unified heterogeneous network $\mathcal{N} = \{\mathcal{V}, \mathcal{E}, \mathcal{R}\} = \{\mathcal{V}_{D} \cup \mathcal{V}_{K}, \mathcal{E}_{D} \cup \mathcal{E}_{K}, \mathcal{R}_{D} \cup \mathcal{R}_{K}\}$, which incorporates both semantic and structural information to enable a more comprehensive characterization of drug relationships. The DDI prediction task is formulated as a classification problem, which can be modeled as either a multi-class or a multi-label setting depending on the requirements. In the multi-class scenario, each drug pair corresponds to a single interaction type, i.e. $\mathcal{R}^{\prime} = \{r \in \mathcal{R}_{D} \mid r = r_{1}\}$. In the multi-label scenario, a drug pair may exhibit multiple interaction types, i.e. $\mathcal{R}^{\prime} = \{r \in \mathcal{R}_{D} \mid r = r_{1}, r_{2}, r_{3}, \ldots \}$. The ultimate goal of this study is to learn a mapping function from the composite network $\mathcal{N}$ that can accurately predict the interaction type(s) between any given pair of drugs.

**Figure 1.**
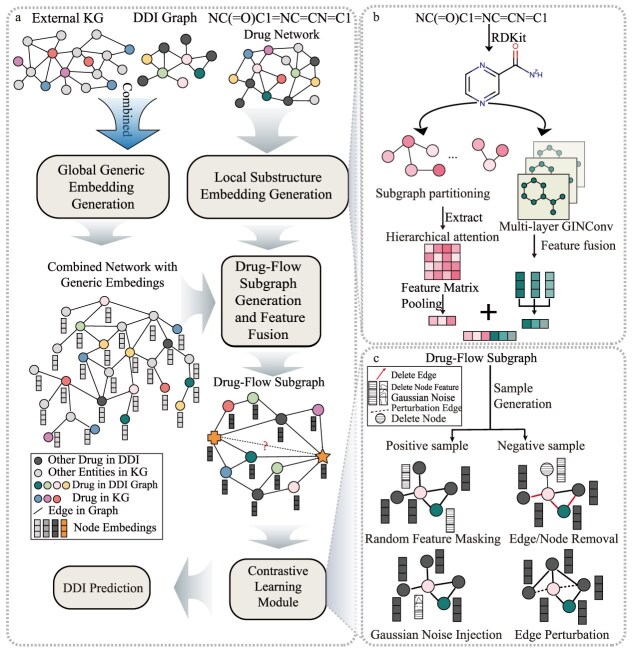
(a) The overall workflow consists of generating a composite KG from an external KG and a DDI network, followed by subgraph extraction and hierarchical feature fusion for DDI prediction. (b) The local substructure embedding module employs hierarchical attention and multi-layer GINConv to extract subgraph-level and atom-level features of the drugs, respectively. (c) A contrastive learning module enhances representation robustness through various graph augmentations (e.g. edge/node removal, noise injection), enforcing consistency between local and global views.

### Overview of MKGFlow-DDI

This section systematically outlines the MKGFlow-DDI model framework. As illustrated in Fig. 1a, the DDI graph is first integrated with an external KG to construct a composite network, enriching all drug and biomedical entity nodes with latent semantic and structural information. Each node receives a unique embedding representation to effectively support downstream tasks. GraphSAGE is subsequently employed to learn general embeddings from the composite network, capturing global semantic information. As depicted in Fig. 1b, a drug substructure extraction module then derives both subgraph-level and atom-level representations. For a given drug pair requiring prediction, corresponding drug subgraphs are extracted from the composite network. Their node features are initialized through weighted aggregation that fuses general embeddings with key substructure embeddings. To fully exploit structural and embedding information while eliminating irrelevant noise, the model performs iterative optimization on each subgraph. During this process, a connectivity-based message-passing mechanism continuously updates both node features and edge weights. Edges falling below a predefined weight threshold are masked, while those exceeding the threshold are promoted to bidirectional connections. Furthermore, when two nodes exhibit substantial embedding similarity after message propagation, a novel bidirectional edge is dynamically established between them. Following multiple iterations, each drug subgraph retains only the most relevant and informative structural components. Finally, as shown in Fig. 1c, a contrastive learning strategy is introduced to further enhance the model’s expressiveness and generalization capability.

### Substructure extraction channel

Drug molecules consist of numerous fundamental substructures that reflect various physicochemical properties. By extracting and integrating these substructures, a more comprehensive and informative feature representation can be obtained [46]. Considering the hierarchical and complex nature of drug molecular structures, we design a MS substructure-aware GNN module. This module employs a dual-channel mechanism (atom-level and subgraph-level) to extract MS structural features, and the representation of key substructures is enhanced through a hierarchical attention fusion mechanism, because conventional graph architectures are inherently limited in capturing chemically meaningful interactions. Specifically, GAT relies primarily on node-level attention without explicitly encoding bond attributes, whereas DiffPool, as a general-purpose pooling strategy, is not tailored to molecular hierarchies, which reduces sensitivity to functional groups and ring systems and limits interpretability.

By contrast, bond information is explicitly incorporated in GINEConv to strengthen atom-level molecular representation, while adaptive hierarchical pooling aligned with chemically relevant substructures is enabled by SAGPool. This combination preserves both chemical specificity and hierarchical modeling, thereby improving representational fidelity and interpretability in molecular graphs. For the atom-level channel, we adopt GINEConv [47] to extract atom-level features of drugs: 


(1) \begin{align*}& \mathbf{h}_{v}^{(l)} = \mathrm{ReLU}\left(\mathbf{W}_{\mathrm{node}}^{(l)} \cdot \mathbf{h}_{v}^{(l-1)} + \sum_{u \in \mathcal{N}(v)} \mathrm{MLP}\left(\mathbf{h}_{u}^{(l-1)} + \mathbf{e}_{uv}\right)\right)\end{align*}

where $\mathbf{W}_{\mathrm{node}}^{(l)}$ is the learnable transformation matrix, $\mathbf{h}_{v}^{(l-1)}$ denotes the feature of node $v$ at layer $l-1$, $\mathbf{e}_{uv}$ is the edge (chemical bond) feature between nodes $u$ and $v$, and $\mathcal{N}(v)$ represents the set of neighbors of node $v$.After obtaining multi-layer atom-level features, we apply a hierarchical attention mechanism to integrate outputs from different layers: 


(2) \begin{align*}& \mathbf{h}_{\mathrm{atom}} = \sum_{l=1}^{L} \alpha^{(l)} \cdot \mathbf{h}_{v}^{(l)}, \quad \alpha^{(l)} = \frac{\exp\left(\mathbf{W}_{2}^\top \tanh\left(\mathbf{W}_{1} \mathbf{h}_{v}^{(l)}\right)\right)}{\sum_{j=1}^{L} \exp\left(\mathbf{W}_{2}^\top \tanh\left(\mathbf{W}_{1} \mathbf{h}_{v}^{(j)}\right)\right)}\end{align*}

where $\mathbf{W}_{1}$ and $\mathbf{W}_{2}$ are learnable parameters, and $\alpha ^{(l)}$ denotes the attention weight assigned to the $l$-th layer, determining its contribution to the final atom-level feature. For the subgraph-level channel, we first decompose each drug molecule into $K$ subgraphs $\{S_{k}\}_{k=1}^{K}$ based on chemical heuristics, and then apply SAGPool to each subgraph: 


(3) \begin{align*}& \mathbf{s}_{k} = \mathrm{READOUT}\left(\left\{\sigma\left(\mathbf{W}^{(Q)} \mathbf{h}_{v}^{(l)}\right) \cdot \mathbf{h}_{v}^{(l)} \mid v \in \mathcal{S}_{k}\right\}\right)\end{align*}

Here, $\mathbf{W}^{(Q)}$ denotes the learnable attention-weight matrix, and $\sigma (\cdot )$ is the activation function, READOUT($\cdot $) aggregates these features to obtain the overall subgraph representation $\mathbf{s}_{k}$. The final subgraph feature is then obtained via a max-pooling operation: 


(4) \begin{align*}& \mathbf{h}_{\mathrm{sub}} = \mathrm{MAXPOOL}\left(\left\{\mathbf{s}_{k}\right\}_{k=1}^{K}\right)\end{align*}

By concatenating the atom-level feature and the subgraph-level feature, we derive the overall drug structural representation: 


(5) \begin{align*}& \mathbf{h}_{\mathrm{drug}} = [\mathbf{h}_{\mathrm{atom}} \parallel \mathbf{h}_{\mathrm{sub}}] \in \mathbb{R}^{2d}\end{align*}

### Composite network general embedding generation

In this work, general embeddings of the composite network are obtained using GraphSAGE [48], where the feature of node $a$ at layer $l+1$ is defined as: 


(6) \begin{align*}& \mathbf{c}_{a}^{(l+1)} = \mathbf{W}_{f}(\sigma\Bigl(\mathbf{W}_{c}^{(l)} \cdot \mathrm{Aggregate}\Bigl({\mathbf{c}_{j}^{(l)}: j \in \varepsilon_{a}}\Bigr) + \omega^{(l)}\Bigr)\Bigr)\end{align*}

where $\mathbf{c}_{j}^{(l)}$ denotes the feature of node $j$ in the composite network at layer $l$, $\mathbf{W}_{\mathrm{f}}\in \mathbb{R}^{2d\times d}$ denotes a learnable weight matrix that projects the features to a ${2d}$ dimensions, $\varepsilon _{a}$ is the set of neighbors of node $a$ at layer $l$, $\mathbf{W}_{c}^{(l)}$ is the learnable weight matrix, $\omega ^{(l)}$ is the bias term, and $\sigma $ is the activation function. The term $\mathrm{Aggregate}\bigl ({\mathbf{c}_{j}^{(l)}: j \in \varepsilon _{a}}\bigr )$ represents an aggregation function that aggregates the features $\mathbf{c}_{j}^{(l)}$ of node $a$’s neighbors in $\varepsilon _{a}$.

### Drug-flow subgraph embedding generation

In the composite network, each drug pair depends on a distinct context, which is represented by entities and relations. For each drug pair $(a, b)$, we construct a drug-specific flow subgraph from the composite network ${\mathcal{N}}{a,b}$. This drug flow subgraph is defined as an undirected subgraph ${S}{a,b}$ that connects $a$ and $b$, with a maximum path length of $P$. Specifically, the construction procedure of the drug flow subgraph ${S}{a,b}$ is illustrated in Algorithm 1.



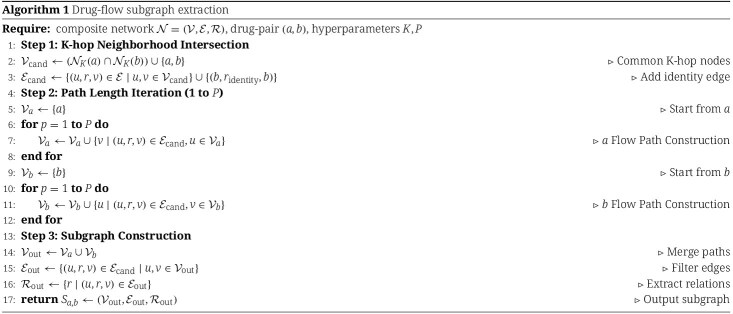



After obtaining the drug flow subgraph, we perform a weighted fusion of the fine-grained substructure features $H_{a,b}^{L}$ and the general embeddings $C_{a,b}^{L}$ obtained from the Composite network to initialize the drug features $\mathbf{S}_{u,v}^{0}=\alpha \cdot \mathbf{H}_{\mathrm{a,b}}^{\mathrm{L}}+(1-\alpha )\cdot \mathbf{C}_{a,b}^{\mathrm{L}}$ in the subgraph,where $\alpha $ denotes the learnable fusion coefficient. Subsequently, we compute the relevance score $\varepsilon _{a,b}^{L}$ by evaluating the similarity between nodes: 


(7) \begin{align*}& \varepsilon_{a,b}^{L} = \rho\left(\left[\exp\left(-\left|S_{u}^{(L-1)} - S_{v}^{(L-1)}\right|\right) \parallel \mathbf{W}_{r}\right]\right)\end{align*}

where $\mathbf{W}_{r}$ denotes the learnable relation embedding for relation $r$, $exp(\cdot )$ is the exponential function, $|\cdot |$ returns the absolute value of each element, and $\rho $ denotes a multilayer perceptron. The learned relevance scores are then used to update the edge connection strengths within the subgraph. 


(8) \begin{align*}& \beta_{a,b}^{(L)} = \sigma\left(\alpha \beta_{a,b}^{(0)} + (1 - \alpha) \varepsilon_{a,b}^{L}\right)\end{align*}

where $\alpha $ is a hyperparameter and $\sigma $ denotes the activation function. $\beta _{a,b}^{(0)}$ is a binary third-order tensor of size $|\tilde{\mathcal{V}}{a,b}| \times |\tilde{\mathcal{V}}{a,b}| \times |\tilde{\mathcal{R}}_{a,b}|$, representing the initial edge connection strength in the drug subgraph. Through iterative updates of the edge strengths, irrelevant information can be effectively filtered out, enabling the model to capture the similarity between drugs. Ultimately, the final drug subgraph embeddings are obtained by aggregating features from neighboring nodes via these weighted connections: 


(9) \begin{align*}& S_{a,b}^{L} = \sigma\left(\varphi\left(\beta_{a,b}^{(L)} S_{a,b}^{L-1} \mathbf{W}_{h}\right)\right)\end{align*}

where $\mathbf{W}_{h}$ is a learnable parameter matrix, $\sigma $ denotes the activation function, and $\varphi $ represents the feature aggregation function applied to each node in the drug subgraph. After $L$ iterations of such updates, the final drug subgraph is produced, and the drug representations are transformed to better capture the predictive logic of the DDI. Finally, the interaction type between $(a, b)$ is predicted as: 


(10) \begin{align*}& \hat{\mathbf{y}}_{a,b} = \rho([\mathbf{h}_{a} \parallel \mathbf{h}_{b}])\end{align*}

where $\rho $ denotes a multilayer perceptron, and $\mathbf{h}_{a}$ and $\mathbf{h}_{b}$ are the final embedding representations of nodes $a$ and $b$ in the drug subgraph, respectively.

### DDI prediction

In this study, the DDI prediction task is formulated as a link prediction problem based on drug subgraphs. Furthermore, the model optimization objective is transformed into a loss minimization problem. For multi-class DDI prediction, we adopt the cross-entropy loss function for optimization: 


(11) \begin{align*}& \ell_{C} = - \sum_{(a,r,b) \in \mathcal{E}D} y{(a,r,b)} \log(\hat{y}_{(a,r,b)})\end{align*}

where $y_{a,b}$ and $\hat{y}_{a,b}$ denote the ground-truth and predicted labels, respectively. For multi-label prediction tasks, we use the binary cross-entropy loss function: 


(12) \begin{align*}& \ell_{BC} = - \sum_{(a,r,b) \in \mathcal{E}D} y{(a,r,b)} \log(\hat{y}{(a,r,b)}) + (1 - y{(a,r,b)}) \log(1 - \hat{y}_{(a,r,b)})\end{align*}

A contrastive loss is introduced to pull similar samples closer and push dissimilar samples apart in the embedding space. This enables the model to preserve more structural information in high-dimensional space, thereby better capturing the potential relationships between drug pairs. Specifically, positive samples are generated by proportionally masking and adding perturbations to node features, whereas negative samples are constructed by randomly removing nodes and edges and introducing perturbations to edge weights. For multiple positive and negative sample pairs, we adopt a novel composite loss computation strategy: 


(13) \begin{align*}& \ell_{\mathrm{NCE}} = - \frac{1}{M} \sum_{p=1}^{M} \log \frac{\exp(z_{p} / \tau)}{\exp(z_{p} / \tau) + \sum_{q=1}^{N} \exp(z_{q} / \tau)}\end{align*}

where $z_{p}$ and $z_{q}$ denote the similarity scores of positive and negative samples, respectively, and $\tau $ is the temperature parameter. The numbers of positive and negative samples are represented by $M$ and $N$, respectively. Finally, the link prediction task is jointly optimized by computing the total loss as follows: 


(14) \begin{align*}& \ell_{\mathrm{total}} = \ell_{\mathrm{label}} + \lambda \ell_{\mathrm{NCE}}\end{align*}

where $\ell _{\mathrm{label}}$ denotes the label loss corresponding to different prediction tasks, and $\lambda $ is a hyperparameter that controls the contribution of the contrastive loss. The flowchart of the algorithm is illustrated in Algorithm 2.



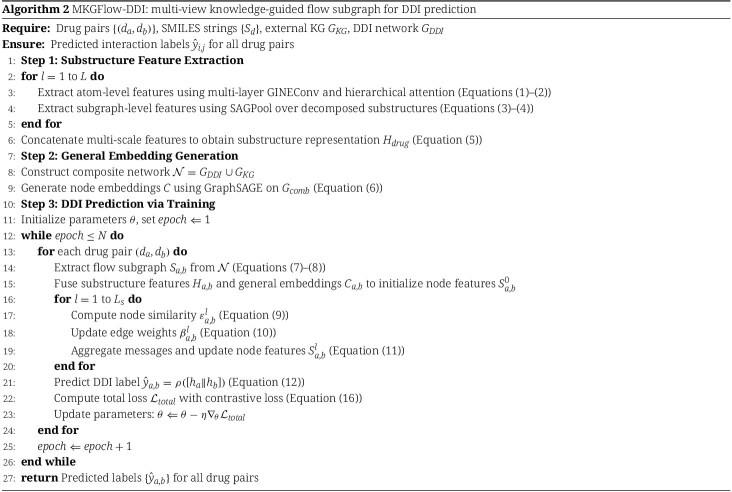



## Experiment results and analysis

This chapter demonstrates the superior performance of MKGFlow-DDI on two benchmark datasets through multiple experiments. We conduct extensive experiments encompassing: (1) performance comparisons with existing state-of-the-art methods; (2) cold-start scenario testing; (3) ablation studies; (4) hyperparameter sensitivity analysis; and (5) interpretability verification and case analysis. Collectively, these experiments demonstrate the model's superior performance in terms of accuracy, robustness, and interpretability.

### Dataset preparation

This study employs two publicly available benchmark datasets: DrugBank [49] and TWOSIDES [50]. DrugBank constitutes a multi-class DDI prediction dataset encompassing pharmacological interactions among 86 drugs, while TWOSIDES represents a multi-label DDI prediction dataset documenting diverse adverse effects between drugs. Hetionet [51] is incorporated as an external KG, serving as an integrated biomedical KG that encapsulates complex biological and medical entities alongside their relationships. For both DrugBank and TWOSIDES, data preprocessing follows the identical pipeline established in SumGNN [17]. Within the DrugBank dataset, edges exhibiting multiple relation types were filtered out due to their rarity relative to the predominance of single-type edges. For TWOSIDES, where each edge may associate with multiple labels, 200 common DDI types were selected through frequency-based curation: all effects were ranked in descending frequency order, with those ranked between 600-800 retained to ensure each effect corresponds to at least 900 drug pairs. This window avoids head-category dominance (inflated by co-prescription patterns) and tail-category instability (sparse samples), ensuring statistically robust assessment of mechanistic modeling. The strategy aligns with established benchmarks (e.g. sumGNN [17]) for discriminative, reproducible evaluation.To prevent information leakage from the external KG Hetionet, edges appearing in both the KG and DDI benchmarks were systematically removed, thereby preserving dataset independence. The processed DDI graphs and external KG were subsequently integrated into a composite network. Experimental fairness was ensured by partitioning DDI triples into training, validation, and test sets at a 7:1:2 ratio. Details of the three datasets are presented in Table 1.

**Table 1:** Statistics of the three benchmark DDI datasets

**Dataset**	**#Nodes**	**#Edges**
DrugBank [49]	1710	192 284
TWOSIDES [50]	645	4 576 287
Hetionet [51]	47 031	2 250 197

### Evaluation metrics

To comprehensively evaluate the performance of the MKGFlow-DDI model, multiple evaluation metrics were employed. For the DrugBank benchmark dataset, which involves multi-class DDI prediction, three metrics were used: accuracy (ACC), macro F1-score (F1), and Cohen’s kappa coefficient. ACC measures the overall classification accuracy, while the macro F1-score balances precision and recall across classes to mitigate the impact of class imbalance. Cohen’s kappa quantifies the agreement between predicted and true labels beyond chance. For the TWOSIDES benchmark dataset, which is suited for multi-label DDI prediction, three metrics were selected: AUROC, AUPRC, and AP@50. AUROC evaluates the model’s overall discriminative ability. AUPRC measures generalization performance on imbalanced data, which is critical when label frequencies vary widely. AP@50 simultaneously considers localization accuracy—measured by the Intersection over Union—and classification accuracy, providing a holistic assessment of practical performance in multi-label settings. To reduce random variation and ensure stability and reliability, each experiment was repeated five times. The final results are reported as the mean and standard deviation of each metric.

### Experimental setup

In the MKGFlow-DDI model, a two-layer GraphSAGE architecture is employed to learn general embeddings for each node in the composite network, with the embedding dimension set to 32. For processing drug subgraphs, the maximum extraction depth is set to 4, and message passing along with edge weight updates are performed through a two-layer subgraph network. To capture drug substructure features, a two-layer network is utilized to extract structural representations at multiple scales.The learning rate is initialized to 0.005, with a weight decay of 0.0001. The model is trained using the Adam optimizer for up to 80 epochs, with early stopping triggered if the validation loss does not improve for 10 consecutive epochs. The batch size is set to 256, and a dropout rate of 0.1 is applied to prevent overfitting. In contrastive learning, positive samples are generated by applying a 20% mask to node features and adding Gaussian noise with a standard deviation $\sigma \in [0.05,0.1]$. Negative samples are constructed by randomly replacing 20% of node features and applying relative perturbations of $\pm $[10%, 30%] to 30% of edge weights. To ensure robustness and reliability, all experiments are repeated five times, with results reported as the mean and standard deviation.

### Baseline

To evaluate the effectiveness of MKGFlow-DDI, we benchmark it against a diverse set of existing methods, ranging from random-walk-based embeddings to KG-aware GNNs. A detailed summary of each baseline is provided in Table 2.

**Table 2:** Summary of baseline models for DDI prediction

**Model**	**Category**	**Description**
GAT-DDI [35]	GNN-based	Applies attention to assign dynamic importance to drug neighbors in the DDI graph.
Decagon [36]	Multi-relational GNN	Models multiple types of DDIs and integrates phenotypic side-effect predictions.
KGNN-DDI [42]	KG-enhanced GNN	Incorporates external biomedical knowledge into GNN aggregation for improved reasoning.
DeepLGF-DDI [45]	KG-enhanced	Combines SMILES-based local features with global KG embeddings using MLP fusion.
AdaProp-DDI [44]	KG-enhanced	Uses adaptive sampling and semantic preservation to reduce noise and improve precision.
SumGNN [17]	KG-enhanced	Extracts summary subgraphs from KG to learn interpretable and informative representations.
KGCNN [19]	KG-enhanced	Captures spatial relationships and higher-level molecular features using capsule aggregators in biomedical graphs.
Know-DDI [20]	KG-enhanced	Utilizes the rich neighborhood information from large biomedical KGs.
LLM-DDI [52]	KG-enhanced	Leverages GPT-derived molecular features within GNNs for predicting drug interactions.

### Performance evaluation on the benchmark dataset

Table 3 compares the performance of MKGFlow-DDI against all baseline methods on both datasets, with the best results highlighted in bold. MKGFlow-DDI consistently outperforms all baselines, demonstrating its effectiveness for DDI prediction. Methods that enhance semantic representations via external KG integration achieve the strongest results, whereas GNN-based approaches without external KGs (e.g. GAT-DDI and Decagon-DDI) perform suboptimally. Although GNNs can propagate and aggregate neighborhood information and employ attention mechanisms to capture complex node embeddings, their inability to leverage semantic information from external KGs limits cross-domain knowledge utilization. Methods combining GNNs with KGs not only improve feature representation but also mitigate issues arising from data scarcity.

**Table 3:** The results evaluated on the DrugBank and TWOSIDES benchmark datasets are presented as mean $\pm $ standard deviation

**Datasets**		**DrugBank**			**TWOSIDES**	
Methods	ACC	F1-score	Cohen’s $\kappa $	AUROC	AUPRC	AP@50
GAT-DDI [35]	79.81$\,\pm\, $1.08	62.05$\,\pm\, $1.92	75.21$\,\pm\, $1.12	86.57$\,\pm\, $0.81	86.27$\,\pm\, $0.79	82.09$\,\pm\, $0.72
Decagon-DDI [36]	82.13$\,\pm\, $0.97	71.27$\,\pm\, $1.54	81.87$\,\pm\, $1.05	87.93$\,\pm\, $0.53	86.44$\,\pm\, $0.57	83.47$\,\pm\, $0.48
KGNN-DDI [42]	85.47$\,\pm\, $0.39	74.52$\,\pm\, $1.18	82.42$\,\pm\, $0.41	88.34$\,\pm\, $0.47	87.13$\,\pm\, $0.51	85.56$\,\pm\, $0.39
DeepLGF-DDI [45]	90.86$\,\pm\, $0.18	85.73$\,\pm\, $0.39	90.08$\,\pm\, $0.22	93.15$\,\pm\, $0.08	92.54$\,\pm\, $0.32	88.67$\,\pm\, $0.15
AdaPro-DDI [44]	89.59$\,\pm\, $0.26	85.44$\,\pm\, $0.43	89.83$\,\pm\, $0.19	92.82$\,\pm\, $0.11	92.21$\,\pm\, $0.34	88.17$\,\pm\, $0.13
SumGNN-DDI [17]	91.24$\,\pm\, $0.14	86.69$\,\pm\, $0.57	90.28$\,\pm\, $0.16	94.31$\,\pm\, $0.09	93.27$\,\pm\, $0.18	89.02$\,\pm\, $0.08
KGCNN [19]	91.31$\,\pm\, $0.13	86.18$\,\pm\, $0.52	90.43$\,\pm\, $0.21	94.01$\,\pm\, $0.10	93.31$\,\pm\, $0.16	88.95$\,\pm\, $0.05
Know-DDI [20]	91.53$\,\pm\, $0.24	93.17$\,\pm\, $0.09	91.89$\,\pm\, $0.11	95.43$\,\pm\, $0.02	94.14$\,\pm\, $0.03	89.54$\,\pm\, $0.03
LLM-DDI [52]	92.28$\,\pm\, $0.19	91.43$\,\pm\, $0.52	92.19$\,\pm\, $0.19	94.43$\,\pm\, $0.14	94.27$\,\pm\, $0.07	89.67$\,\pm\, $0.04
**MKGFlow-DDI**	**94.28$\,\pm\, $0.13**	**93.37$\,\pm\, $0.49**	**93.11$\,\pm\, $0.09**	**95.96$\,\pm\, $0.06**	**95.18$\,\pm\, $0.04**	**90.62$\,\pm\, $0.03**

KGNN-DDI underperforms because noise in the KG disrupts the capture of drug relationships and their neighborhoods, reducing prediction accuracy. SumGNN-DDI addresses this by integrating the DDI network with external KGs to construct a composite network and encoding semantic and structural information through subgraph extraction after node embedding, thereby enhancing resilience to noise. Subsequent methods such as Know-DDI and LMM-DDI better capture higher-order features of molecular graph structures but often overlook intrinsic drug node features, relying heavily on network topology. MKGFlow-DDI overcomes these limitations by fusing drug substructure features within subgraphs, filtering noise through updated relevance scores, and promoting robust information exchange via a subgraph contrastive learning module. These enhancements account for its superior performance relative to existing methods.

### Cold start experiment

To assess the applicability of the model to DDI prediction in real-world drug discovery, its generalization under cold-start conditions was examined. This setting simulates the continual emergence of new drugs and demands reliable prediction for compounds unseen during training. Two strict drug-level cold-start protocols were designed to evaluate MKGFlow-DDI’s robustness: S1 (single-drug cold-start) and S2 (dual-drug cold-start). In S1 each test pair contains one unseen drug; in S2 both drugs in the test pair are unseen. All experiments used the same metrics and baselines as the main experiments to ensure fair comparison.

As shown in Table 4, performance declined across all models under cold-start conditions, underscoring the difficulty of this task. Under the S1 setting, the mean F1 score of baseline models decreased from 81.83% in the warm-start regime to 61.53%, indicating a substantial degradation. Notably, MKGFlow-DDI maintained strong and stable performance, ranking first in ACC, F1 score, and Cohen’s $\kappa $, and significantly outperforming the strongest baseline. These results indicate that MKGFlow-DDI effectively handles common cold-start scenarios involving a single novel drug and remains highly reliable even under the extreme setting where both drugs are unseen. Further comparisons showed that, although all models exhibited performance drops, the decline of MKGFlow-DDI was markedly smaller, suggesting greater robustness and adaptability of its representation learning and inference mechanisms to entirely unseen entities.

**Table 4:** The cold-start experimental results on the DrugBank benchmark dataset are presented as mean $\pm $ standard deviation

	DrugBank					
Methods	ACC	F1-score	Cohen’s $\kappa $	ACC	F1-score	Cohen’s $\kappa $
GAT-DDI [35]	68.21$\,\pm\, $1.51	50.13$\,\pm\, $2.16	65.24$\,\pm\, $0.18	48.44$\,\pm\, $2.81	36.61$\,\pm\, $2.44	48.53$\,\pm\, $0.28
Decagon-DDI [36]	71.53$\,\pm\, $1.22	55.34$\,\pm\, $1.87	68.55$\,\pm\, $0.15	52.73$\,\pm\, $2.54	45.23$\,\pm\, $2.69	52.34$\,\pm\, $0.24
KGNN-DDI [42]	73.87$\,\pm\, $1.04	58.63$\,\pm\, $1.65	71.07$\,\pm\, $0.12	55.37$\,\pm\, $2.38	48.84$\,\pm\, $2.06	55.84$\,\pm\, $0.25
DeepLGF-DDI [45]	76.40$\,\pm\, $0.89	62.12$\,\pm\, $1.36	73.48$\,\pm\, $0.10	58.18$\,\pm\, $2.16	51.53$\,\pm\, $2.23	59.23$\,\pm\, $0.23
AdaPro-DDI [44]	75.96$\,\pm\, $0.97	61.84$\,\pm\, $1.41	73.29$\,\pm\, $0.11	59.86$\,\pm\, $1.65	52.97$\,\pm\, $2.12	58.74$\,\pm\, $0.21
SumGNN-DDI [17]	78.28$\,\pm\, $0.76	65.36$\,\pm\, $1.24	75.86$\,\pm\, $0.09	60.73$\,\pm\, $1.83	53.63$\,\pm\, $1.83	62.14$\,\pm\, $0.19
KGCNN [19]	77.86$\,\pm\, $0.83	64.76$\,\pm\, $1.38	75.92$\,\pm\, $0.10	60.24$\,\pm\, $1.68	52.91$\,\pm\, $1.54	61.56$\,\pm\, $0.17
Know-DDI [20]	79.54$\,\pm\, $0.62	67.16$\,\pm\, $1.16	77.41$\,\pm\, $0.08	62.35$\,\pm\, $1.57	54.84$\,\pm\, $1.60	64.31$\,\pm\, $0.16
LLM-DDI [52]	80.23$\,\pm\, $0.50	68.44$\,\pm\, $1.04	78.62$\,\pm\, $0.07	63.52$\,\pm\, $1.43	56.20$\,\pm\, $1.45	65.81$\,\pm\, $0.18
**MKGFlow-DDI**	**84.75$\,\pm\, $0.44**	**73.36$\,\pm\, $0.92**	**82.93$\,\pm\, $0.12**	**70.46$\,\pm\, $0.83**	**64.94$\,\pm\, $1.26**	**69.48$\,\pm\, $0.15**

MKGFlow-DDI’s generalization arises from deep fusion and inductive design. Atom-level substructures were fused with high-level knowledge-graph semantics to produce hierarchical, transferable representations grounded in intrinsic chemical and biological properties, affording interpretability for unseen drugs. The GNN’s inductive capacity enabled effective representation inference for novel compounds via molecular topology and the KG neighborhood. A contrastive learning module enhanced discriminability and robustness by pulling similar representations together and separating dissimilar ones. This yielded precise embeddings for novel drugs based on structural and semantic similarity. Collectively, these mechanisms enabled MKGFlow-DDI to generalize to novel-drug prediction in real-world settings, surpassing baseline methods.

### Ablation study

To investigate the contribution of each key component in MKGFlow-DDI, several ablated variants were constructed for comparative analysis. Specifically, the role of substructure-level encoding was evaluated by removing the substructure learning module and relying solely on general node embeddings from the composite network [MKGFlow-DDI(SL)]. The impact of representation robustness was assessed by disabling the contrastive learning objective and training exclusively with standard classification loss [MKGFlow-DDI(CL)]. The effect of semantic edge weighting was tested by setting all edge weights in the drug-flow subgraph to unity, effectively eliminating similarity-aware propagation [MKGFlow-DDI(KSW)]. Additionally, varying degrees of knowledge incompleteness were simulated by randomly removing 20%, 50%, and 80% of entity pairs from the external biomedical knowledge graph prior to composite network construction [denoted MKGFlow-DDI(−20% KG), MKGFlow-DDI(−50% KG), and MKGFlow-DDI(−80% KG), respectively]. These ablations enable systematic examination of each design component’s contribution and robustness under controlled perturbations.

**Figure 2.**
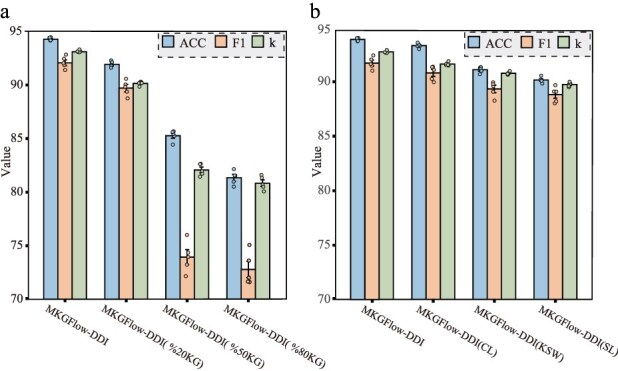
Performance of MKGFlow-DDI variants on DrugBank. The x-axis shows key hyperparameter values, and the y-axis reports corresponding performance scores; different colored lines distinguish the evaluation metrics.

As shown in Fig. 2, all MKGFlow-DDI variants exhibit performance degradation relative to the complete model, confirming each module’s importance for DDI prediction. Figure 2a demonstrates progressive performance decline as KG removal proportion increases, validating the KG’s efficacy in model learning. Performance degradation becomes most substantial at 80% removal, where the residual 20% of entities provide insufficient contextual and topological information. Figure 2b reveals that MKGFlow-DDI(SL) retains KG contextual information yet exhibits significant performance deterioration. While capable of learning from the rich KG, this variant loses molecular structural comprehension. MKGFlow-DDI(KSW) similarly underperforms, as ablating edge weight updating prevents modeling relationship strengths within the graph, yielding suboptimal information propagation. Finally, while MKGFlow-DDI(CL) exhibits slightly reduced performance, its contrastive learning module promotes more discriminative feature extraction by contrasting positive and negative samples, ultimately improving model robustness—a finding further supported by the hyperparameter sensitivity analysis.

### Hyperparameter sensitivity analysis

This study conducts hyperparameter sensitivity analysis using the DrugBank dataset. While maintaining fixed values for other parameters, we systematically examine the influence of key hyperparameters on MKGFlow-DDI performance: embedding dimension $d$, drug-flow subgraph size $p$, combination graph network layer count $L_{c}$, and subgraph network layer count $L_{s}$.

**Figure 3.**
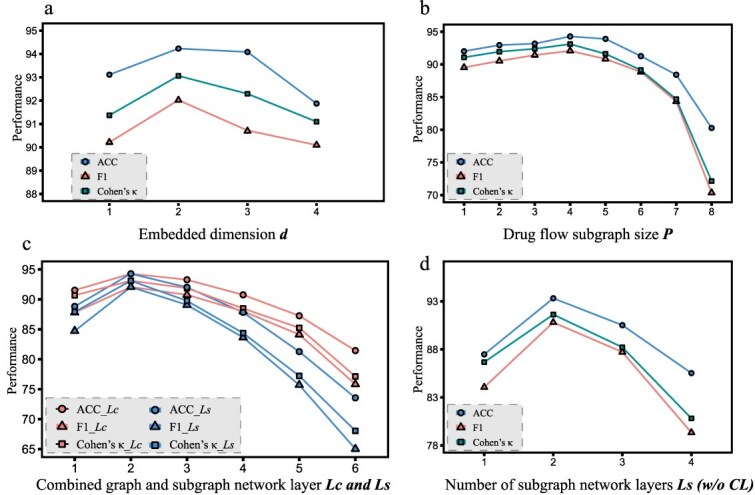
Performance of MKGFlow-DDI variants on the DrugBank benchmark dataset.

Figure 3a illustrates optimal model performance at embedding dimension $d=32$. Insufficiently small dimensions compromise the capture of complex data features, degrading performance, while excessively large dimensions induce overfitting or computational overhead, similarly impairing results. Figure 3b indicates peak performance at subgraph size $p=4$. Beyond $p>7$, however, performance exhibits unstable decline due to exponential subgraph growth that amplifies noise within the drug-flow subgraph. Figure 3c reveals optimal performance for both $L_{c}$ and $L_{s}$ at two layers. Deviations in either direction degrade results: excessive layers introduce prediction-irrelevant data, while insufficient layers underutilize contextual information from the external KG. Notably, performance degrades more rapidly with increasing $L_{s}$ than $L_{c}$, attributable to the combination network’s prior multi-layer aggregation having already captured broader neighborhood information for each drug in the drug-flow subgraph.

Extending this analysis, we evaluate the contrastive learning module’s contribution. Removing this component and progressively increasing $L_{s}$ reveals substantial performance deterioration by $L_{s}=4$ (Fig. 3d), confirming the module’s critical role in enhancing both predictive performance and generalization capability.

### Interpretability experiments

In this study, MKGFlow-DDI utilizes similarity scores to evaluate the importance of edges within the subgraph, thereby predicting DDIs. By analyzing the weight relationships of edges in the subgraph, interpretable paths can be derived to reveal the types of interactions between drugs.

**Figure 4.**
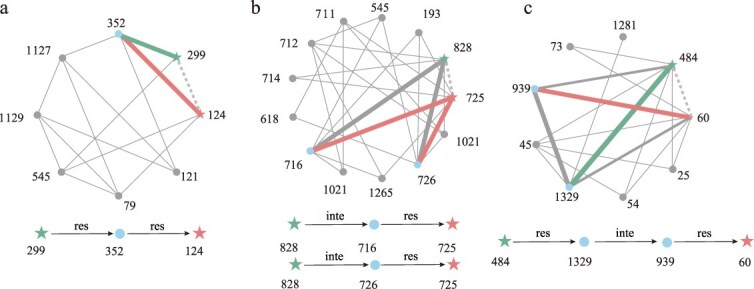
Path-based explainable graph, where "res" and "inte" denote resemblance and interaction, respectively. All nodes are drug entities; star-shaped nodes mark the target drug pair. Gray solid lines indicate weaker similarity edges, while red and green solid lines indicate stronger similarity relationships.

Figure 4 illustrates the relationships between nodes and edges within the subgraphs for three drug pairs, where the thickness of the edges indicates their importance. As shown in Fig. 4a, nodes (299) and (124) exhibit strong similarity to the intermediate node (352), and a DDI relationship exists between nodes (124) and (352). Consequently, it can be inferred that this drug pair may produce similar effects when used concurrently. MKGFlow-DDI does not rely solely on a single path for DDI prediction. Figure 4b demonstrates a scenario where DDIs are predicted via multiple interpretable paths. Drug nodes (726) and (716) both interact with node (828). Furthermore, MKGFlow-DDI identifies high similarity between node (725) and both nodes (716) and (726), leading to the inference that a DDI may also occur between nodes (725) and (828). Additionally, MKGFlow-DDI can perform reasoning through indirect DDI inference paths. As depicted in Fig. 4c, strong connection strengths exist between nodes (484) and (1329), and between nodes (60) and (939). By learning the reaction type between nodes (939) and (1329), the model further infers that a similar DDI relationship might exist between nodes (484) and (60).

In summary, these interpretable paths are not only reasonable but also align with the model’s predictive objectives. By continuously updating edge weights, MKGFlow-DDI learns subgraphs more tightly coupled with the target prediction relationship. This facilitates the identification of critical paths, and precisely these paths reflect the correlation between the types of interactions among drug nodes and their interpretability.

### Case study

**Figure 5.**
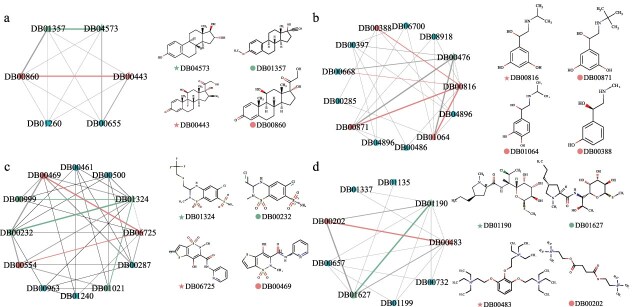
Case analysis graph based on drug structures. All nodes represent drugs, with the star-shaped node pair (connected by gray dashed lines) marking the target pair for prediction. Gray solid lines indicate weaker similarity edges, while red and green solid lines indicate stronger similarity edges, with line width encoding edge importance. Panels (a)–(d) show comparative diagrams of key drug structures.

To further evaluate the reliability of the interpretable paths generated based on similarity scores and validate the practical utility of MKGFlow-DDI in real-world scenarios, this study conducted a case analysis experiment. As illustrated in Fig. 5, we examined the SMILES structures of the drug pairs within the drug-flow subgraph and the two drugs corresponding to the highest connection weight scores, followed by a detailed analysis. Taking Fig. 5a as an example, DB01357 (Mestranol) is a synthetic estradiol used in oral contraceptives and in the treatment of other disorders of the female reproductive system, such as dysmenorrhea and dysfunctional uterine bleeding. DB00860 (Prednisolone) is a glucocorticoid similar to cortisol, possessing anti-inflammatory, immunosuppressive, antineoplastic, and vasoconstrictive properties. Relevant studies indicate that the co-administration of Prednisolone and Mestranol can increase serum concentrations. MKGFlow-DDI identified a strong similarity path between DB01357 (Mestranol) and DB00860 (Prednisolone), involving DB04573 (Estriol) and DB00443 (Betamethasone), and further inferred that a similar DDI might also exist between Estriol and Betamethasone. Estriol is a weak estrogen used to treat vaginal dryness and other symptoms of estrogen deficiency, such as vaginitis and vulvar pruritus. Betamethasone is a systemic corticosteroid employed to reduce inflammation in a wide range of conditions, including allergic states, dermatological diseases, gastrointestinal disorders, and hematological conditions. To further verify this inference, we analyzed the molecular structures of these drugs. As shown in the molecular structure diagrams on the right side of Fig. 5a, Estriol and Mestranol share the same fundamental core structure, both featuring a tetracyclic ring system fused to a six-membered ring. Similarly, Betamethasone and Prednisolone exhibit highly similar ring structures and functional groups. These structural similarities suggest that they may exert analogous biological effects. More significantly, we identified supporting evidence in DrugBank and related literature [53] indicating that the concomitant use of Estriol and Betamethasone may also lead to increased serum concentrations, thereby further validating the reliability of MKGFlow-DDI’s inference.

Through this case analysis, we demonstrate MKGFlow-DDI’s capability of capturing potential DDIs. The results also highlight its effectiveness and reliability in practical applications. Furthermore, the case analysis provides additional evidence of the model’s advantages in DDI prediction. It not only accurately predicts potential DDIs based on molecular structures and similarity information but also provides interpretable paths that offer valuable clinical insights, helping researchers and healthcare professionals make evidence-based decisions about drug co-administration. Consequently, MKGFlow-DDI shows great potential for application in biomedical research and holds significant practical value.

## Conclusions and discussion

This study presents MKGFlow-DDI, a multi-view knowledge-guided framework for explainable DDI prediction that unifies biomedical KGs with molecular substructures. By dynamically constructing drug-flow subgraphs through a dual-channel architecture, our approach captures MS structural and semantic representations. Comprehensive evaluations on DrugBank and TWOSIDES benchmarks demonstrate significant performance improvements, particularly in cold-start scenarios with unseen drugs, where MKGFlow-DDI consistently outperforms state-of-the-art baselines across multiple metrics.

The framework introduces four synergistic innovations: hierarchical feature extraction via GINEConv and SAGPool enables atomic-to-subgraph level encoding; GraphSAGE-based fusion integrates local embeddings with global knowledge semantics; adaptive edge reweighting enhances subgraph fidelity through noise suppression and biological interaction amplification; and contrastive learning regularization improves robustness under data scarcity. Crucially, semantic path reasoning links predictions to interpretable subgraph trajectories elucidating pharmacological mechanisms, with ablation studies confirming the critical synergy between structural granularity, contextual integration, and representation alignment.

While establishing a principled foundation for interpretable DDI prediction, several research avenues merit exploration. Current KG dependencies could be mitigated by incorporating complementary modalities such as gene expression profiles or metabolic pathways. Negative sampling strategies warrant enhancement through adversarial perturbations or domain-aware augmentation. With the rapid advancement of artificial intelligence, existing frameworks for interpretability evaluation still face certain limitations. Moving forward, we will focus on “quantitative interpretability assessment” as a core direction, aiming to overcome the current bottleneck of insufficient quantitative evidence in model explanation mechanisms and to systematically enhance the verifiability and reliability of the model’s reasoning process. Most critically, developing efficient continual learning mechanisms for evolving pharmacological landscapes remains essential for real-world deployment.

MKGFlow-DDI’s capacity to dynamically fuse domain knowledge with molecular representations while providing mechanistic insights positions it as a transformative approach for computational pharmacovigilance and precision therapy. The framework’s adaptability to novel drug entities and biologically grounded explanations addresses fundamental requirements in next-generation drug safety surveillance.

**Key Points**

- We propose MKGFlow-DDI, a novel multi-view framework that integrates drug substructures, biomedical knowledge graphs, and flow-based subgraph reasoning to enable interpretable and mechanistically grounded DDI prediction.
- We design a dual-channel encoder with contrastive subgraph learning and semantic edge filtering to improve representation quality and enhance generalization.
- MKGFlow-DDI achieves state-of-the-art results on DDI benchmarks and generates biologically plausible paths supporting mechanistic interpretation and clinical translation.

## Supplementary Material

Supplementary_elag006

## Data Availability

The data and source code underlying this study are publicly available. The implementation of *MKGFlow_DDI* can be found at https://github.com/ZhangLab312/MKGFlow_DDI.git. The benchmark datasets used in this work include DrugBank (https://go.drugbank.com/), TWOSIDE (https://nsides.io/), and Hetionet (https://het.io/).

## Notes

**Yugui Xu.** He is working toward an M.S. degree at Chengdu University of Information Technology, Chengdu, China. His research interests include machine learning and bioinformatics.

**Zhigan Zhou.** He is working toward an M.S. degree at Chengdu University of Information Technology, Chengdu, China. His research interests include machine learning and bioinformatics.

**Hao Yuan.** He received the M.S. degree from Chengdu University of Information Technology, Chengdu, China, in 2025. His research interests include machine learning and bioinformatics.

**Siqi Liu.** She is working toward an M.S. degree at Chengdu University of Information Technology, Chengdu, China. Her research interests include machine learning and data mining.

**Quan Zou.** He received the Ph.D. from Harbin Institute of Technology in 2009 and is now a Professor at the University of Electronic Science and Technology of China. His research focuses on bioinformatics, machine learning, and parallel computing.

**Yongqing Zhang.** He received the Ph.D. degree from Sichuan University, Chengdu, China, in 2016. He is a Professor at Chengdu University of Information Technology, Chengdu, China. His research interests include machine learning and bioinformatics.

**Wenqian Zhang.** He received Doctor of Medicine, Deputy Director of Thoracic Surgery at Beijing Chaoyang Hospital, graduated from Xiangya Medical College of Central South University and Capital Medical University, and holds a postdoctoral position at the University of California, San Francisco (UCSF) in the United States. My main research areas are pulmonary nodules, lung cancer, and bioinformatics.

## References

[1] Ren Z, Zeng X, Lao Y et al. Predicting rare drug-drug interaction events with dual-granular structure-adaptive and pair variational representation. *Nat Commun* 2025; 16:3997. 10.1038/s41467-025-59431-9. 40301328 PMC12041321

[2] Li X, Rao K, Chen C et al. A cell type and state specific gene regulation network inference method for immune regulatory analysis. *NPJ Syst Biol Appl* 2025; 11:94. 10.1038/s41540-025-00564-4. 40804307 PMC12350830

[3] Ong JCL, Chen MH, Ng N et al. A scoping review on generative AI and large language models in mitigating medication related harm. *npj Digit Med* 2025; 8:182. 10.1038/s41746-025-01565-7. 40155703 PMC11953325

[4] Li X, Chen C, Li Y et al. A cell-cell interaction network based method for identifying lymph node metastasis and characterizing lung cancer progression. *IEEE Trans Comput Biol Bioinform* 2025; 22:2750–63. 10.1109/TCBBIO.2025.3600428.

[5] Del Re , Roncato R, Argentiero A et al. Clinical relevance and methodological approach for the assessment of drug–drug interactions in cancer patients: a position statement from the Italian Association of Medical Oncology (AIOM) and the Italian Society of Pharmacology (SIF). *ESMO Open* 2025; 10:105119. 10.1016/j.esmoop.2025.105119. 40499461 PMC12182795

[6] Li X, Hua Y, Liu H et al. Peripheral blood multimodal integration via cross-attention for cancer immune profiling. *BMC Cancer* 2025; 25:1523. 10.1186/s12885-025-14969-1. 41053622 PMC12502316

[7] Schroeter T, Lapham K, Varma MVS et al. Positioning enzyme-and transporter-based precipitant drug–drug interaction studies in drug design. *J Med Chem* 2025; 68:1021–32. 10.1021/acs.jmedchem.4c02629. 39762221

[8] Gottlieb A, Stein GY, Oron Y et al. INDI: a computational framework for inferring drug interactions and their associated recommendations. *Mol Syst Biol* 2012; 8:592. 22806140 10.1038/msb.2012.26 PMC3421442

[9] Li P, Huang C, Yingxue F et al. Large-scale exploration and analysis of drug combinations. *Bioinformatics* 2015; 31:2007–16. 10.1093/bioinformatics/btv080. 25667546

[10] Jianbo Qiao X, Guo JJ, Wang D et al. Taco-DDI: accurate prediction of drug-drug interaction events using graph transformers and dynamic co-attention matrices. *Neural Netw* 2025; 189:107655. 10.1016/j.neunet.2025.107655. 40446573

[11] Ryu JY, Kim HU, Lee SY. Deep learning improves prediction of drug–drug and drug–food interactions. *Proc Natl Acad Sci* 2018; 115:E4304–11. 29666228 10.1073/pnas.1803294115 PMC5939113

[12] Nyamabo AK, Hui Y, Liu Z et al. Drug–drug interaction prediction with learnable size-adaptive molecular substructures. *Brief Bioinform* 2022; 23:bbab441. 10.1093/bib/bbab441. 34695842

[13] Lin S, Wang Y, Zhang L et al. MDF-SA-DDI: predicting drug–drug interaction events based on multi-source drug fusion, multi-source feature fusion and transformer self-attention mechanism. *Brief Bioinform* 2022; 23:bbab421. 34671814 10.1093/bib/bbab421

[14] Niu D, Lei X, Pan S et al. SRR-DDI: a drug–drug interaction prediction model with substructure refined representation learning based on self-attention mechanism. *Knowl-Based Syst* 2024; 285:111337. 10.1016/j.knosys.2023.111337.

[15] Zhang X, Xiang H, Yang X et al. Dual-view learning based on images and sequences for molecular property prediction. *IEEE J Biomed Health Inform* 2023; 28:1564–74. 10.1109/JBHI.2023.3347794. 38153823

[16] Karim MR, Cochez M, Jares JB et al. Drug-drug interaction prediction based on knowledge graph embeddings and convolutional-lstm network. In: Proceedings of the 10th ACM International Conference on Bioinformatics, Computational Biology and Health Informatics, pp. 113–123, 2019.

[17] Yue Y, Huang K, Zhang C et al. SumGNN: multi-typed drug interaction prediction via efficient knowledge graph summarization. *Bioinformatics* 2021; 37:2988–95. 10.1093/bioinformatics/btab207. 33769494 PMC10060701

[18] Hong Y, Luo P, Jin S et al. LaGAT: link-aware graph attention network for drug–drug interaction prediction. *Bioinformatics* 2022; 38:5406–12. 10.1093/bioinformatics/btac682. 36271850 PMC9750103

[19] Xiaorui S, Zhao B, Li G et al. Knowledge graph neural network with spatial-aware capsule for drug-drug interaction prediction. *IEEE J Biomed Health Inform* 2024; 29:1771–81. 10.1109/JBHI.2024.3419015. 38917286

[20] Wang Y, Yang Z, Yao Q. Accurate and interpretable drug-drug interaction prediction enabled by knowledge subgraph learning. *Commun Med* 2024; 4:59. 10.1038/s43856-024-00486-y. 38548835 PMC10978847

[21] Hua Y, Na H, Li Z et al. A scoping review of large language models for generative tasks in mental health care. *npj Digit Med* 2025; 8:230. 10.1038/s41746-025-01611-4. 40307331 PMC12043943

[22] Othman ZK, Ahmed MM, Okesanya OJ et al. Advancing drug discovery and development through GPT models: a review on challenges, innovations and future prospects. *Intell-Based Med* 2025; 11:100233. 10.1016/j.ibmed.2025.100233.

[23] Cheng F, Zhao Z. Machine learning-based prediction of drug–drug interactions by integrating drug phenotypic, therapeutic, chemical, and genomic properties. *J Am Med Inform Assoc* 2014; 21:e278–86. 10.1136/amiajnl-2013-002512. 24644270 PMC4173180

[24] Zhang P, Wang F, Jianying H et al. Label propagation prediction of drug-drug interactions based on clinical side effects. *Sci Rep* 2015; 5:12339. 10.1038/srep12339. 26196247 PMC5387872

[25] Perozzi B, Al-Rfou R, Skiena S. DeepWalk: online learning of social representations. In: Proceedings of the 20th ACM SIGKDD International Conference on Knowledge Discovery and Data Mining, Association for Computing Machinery, pp. 701–710, 2014.

[26] Grover A, Leskovec J. node2vec: scalable feature learning for networks. In: Proceedings of the 22nd ACM SIGKDD International Conference on Knowledge Discovery and Data Mining, Association for Computing Machinery, pp. 855–864, 2016. 10.1145/2939672.2939754 PMC5108654 27853626

[27] Meng L, He Y, Sun C et al. Learning personalized drug features and differentiated drug-pair interaction information for drug–drug interaction prediction. *Neural Netw* 2025; 181:106828. 10.1016/j.neunet.2024.106828. 39490025

[28] Xiong Z, Liu S, Huang F et al. Multi-relational contrastive learning graph neural network for drug-drug interaction event prediction. In: *Proceedings of the AAAI Conference on Artificial Intelligence*, AAAI Press, Vol. 37, 2023; pp. 5339–47. 10.1609/aaai.v37i4.25665.

[29] Zhou Z, Wei J, Liu M et al. AnomalGRN: deciphering single-cell gene regulation network with graph anomaly detection. *BMC Biol* 2025; 23:73. 10.1186/s12915-025-02177-z. 40069807 PMC11900578

[30] Wang J-C, Chen Y-J, Zou Q. GRACE: unveiling gene regulatory networks with causal mechanistic graph neural networks in single-cell RNA-sequencing data. *IEEE Trans Neural Netw Learn Syst*, 2025;36:9005–9017. 10.1109/TNNLS.2024.3412753. 38896510

[31] Wang J, Chen Y, Zou Q. Inferring gene regulatory network from single-cell transcriptomes with graph autoencoder model. *PLoS Genet* 2023; 19:e1010942. 10.1371/journal.pgen.1010942. 37703293 PMC10519590

[32] Zhong Y, Zheng H, Xiaoming Chen Y et al. DDI-GCN: drug-drug interaction prediction via explainable graph convolutional networks. *Artif Intell Med* 2023; 144:102640. 10.1016/j.artmed.2023.102640. 37783544

[33] Kipf TN, Welling M. Semi-supervised classification with graph convolutional networks. arXivpreprintarXiv:1609.02907. OpenReview.net, 2017:1–14.

[34] Nyamabo AK, Hui Y, Shi J-Y. SSI–DDI: substructure–substructure interactions for drug–drug interaction prediction. *Brief Bioinform* 2021; 22:bbab133. 33951725 10.1093/bib/bbab133

[35] Velickovic P, Cucurull G, Casanova A et al. Graph attention networks. *stat* 2017; 1050:10–48550.

[36] Zitnik M, Agrawal M, Leskovec J. Modeling polypharmacy side effects with graph convolutional networks. *Bioinformatics* 2018; 34:i457–66. 10.1093/bioinformatics/bty294. 29949996 PMC6022705

[37] Huang K, Xiao C, Glass LM et al. SkipGNN: predicting molecular interactions with skip-graph networks. *Sci Rep* 2020; 10:21092. 33273494 10.1038/s41598-020-77766-9 PMC7713130

[38] Peng W, Chen C, Dai W et al. Predicting clinical anticancer drug response of patients by using domain alignment and prototypical learning. *IEEE J Biomed Health Inform* 2024; 29:1534–45. 10.1109/JBHI.2024.3462811. 39292588

[39] Peng W, Lin J, Dai W et al. Hierarchical graph representation learning with multi-granularity features for anti-cancer drug response prediction. *IEEE J Biomed Health Inform* 2024; 29:7839–50. 10.1109/JBHI.2024.3492806. 39504283

[40] Peng W, Liu H, Dai W et al. Predicting cancer drug response using parallel heterogeneous graph convolutional networks with neighborhood interactions. *Bioinformatics* 2022; 38:4546–53. 10.1093/bioinformatics/btac574. 35997568

[41] Wang Y, Zhai Y, Ding Y et al. SBSM-Pro: support bio-sequence machine for proteins. *Sci China Inf Sci* 2024; 67:212106. 10.1007/s11432-024-4171-9.

[42] Lin X, Quan Z, Wang Z-J et al. KGNN: knowledge graph neural network for drug-drug interaction prediction. In: *IJCAI, International Joint Conferences on Artificial Intelligence Organization*, Vol. 380, 2020; pp. 2739–45.

[43] Xiaorui S, Lun H, You Z et al. Attention-based knowledge graph representation learning for predicting drug-drug interactions. *Brief Bioinform* 2022; 23:bbac140. 10.1093/bib/bbac140. 35453147

[44] Zhang Y, Zhou Z, Yao Q et al. Adaprop: Learning adaptive propagation for graph neural network based knowledge graph reasoning. In: Proceedings of the 29th ACM SIGKDD Conference on Knowledge Discovery and Data Mining, Association for Computing Machinery, pp. 3446–3457, 2023.

[45] Ren Z-H, You Z-H, Chang-Qing Y et al. A biomedical knowledge graph-based method for drug–drug interactions prediction through combining local and global features with deep neural networks. *Brief Bioinform* 2022; 23:bbac363. 10.1093/bib/bbac363. 36070624

[46] Chen M, Gong X, Pan S et al. Unified knowledge-guided molecular graph encoder with multimodal fusion and multi-task learning. *Neural Netw* 2025; 184:107068. 10.1016/j.neunet.2024.107068. 39732065

[47] Hu W, Liu B, Gomes J et al. Strategies for pre-training graph neural networks. International Conference on Learning Representations, 2020, 1–22. arXivpreprintarXiv:1905.12265.

[48] Liu J, Ong GP, Chen X. Graphsage-based traffic speed forecasting for segment network with sparse data. *IEEE Trans Intell Transp Syst* 2020; 23:1755–66. 10.1109/TITS.2020.3026025.

[49] Wishart DS, Feunang YD, Guo AC et al. DrugBank 5.0: a major update to the drugbank database for 2018. *Nucleic Acids Res* 2018; 46:D1074–82. 10.1093/nar/gkx1037. 29126136 PMC5753335

[50] Tatonetti NP, Ye PP, Daneshjou R et al. Data-driven prediction of drug effects and interactions. *Sci Transl Med* 2012; 4:125ra31–1. 10.1126/scitranslmed.3003377. PMC3382018 22422992

[51] Himmelstein DS, Baranzini SE. Heterogeneous network edge prediction: a data integration approach to prioritize disease-associated genes. *PLoS Comput Biol* 2015; 11:1004259. 10.1371/journal.pcbi.1004259. PMC4497619 26158728

[52] Li D, Yang Y, Cui Z et al. LLM-DDI: leveraging large language models for drug-drug interaction prediction on biomedical knowledge graph. *IEEE J Biomed Health Inform* 2026;30:773–781. 10.1109/JBHI.2025.3585290. 40601466

[53] Seidegård J, Simonsson M, Edsbäcker S. Effect of an oral contraceptive on the plasma levels of budesonide and prednisolone and the influence on plasma cortisol. *Clin Pharmacol Ther* 2000; 67:373–81. 10.1067/mcp.2000.105762. 10801246

