# Supplementary material for: Multi-view knowledge-guided flow subgraphs with substructure initialization for explainable DDI prediction
Source: Brief Funct Genomics. 2026 Jul 1;25:elag006. doi: 10.1093/bfgp/elag006 (PMC13320273; doi:10.1093/bfgp/elag006)
Supplement: Supplementary_elag006 [file supplementary_elag006.pdf]

Supplementary Table 1: Symbols and description s in MKGFlow-DDI.

Symbol	Description
N_D	Drug-drug interaction network containing interaction information
N_K	External biomedical knowledge graph with rich biological information
N	Unified heterogeneous network integrating DDI and knowledge graphs
$h_v^{(l)}$	Atom-level chemical feature of node v at layer l
W	Represents all learnable (trainable) matrices
e_{uv}	Chemical bond feature between nodes u and v
$N(v)$	Set of neighbors of node v
$\alpha^{(l)}$	Attention weight assigned to the l-th layer
w_a, W_a	Parameters for hierarchical attention mechanism
S_k	Subgraph representation for subgraph k
$\sigma(\cdot)$	Activation function for subgraph representation
h_{atom}	Final atom-level feature after hierarchical attention
h_{sub}	Final subgraph feature after max-pooling
h_{drug}	Overall drug structural representation
α	Learnable fusion coefficient for substructure and general embeddings
$\varepsilon_{a,b}^L$	Similarity-based relevance score for edges
$\beta_{a,b}$	Edge connection strength in subgraph
L	Number of iterations for edge refinement
$S_{a,b}^L$	Final drug subgraph embeddings after iterations
h_a, h_b	Final embedding representations of nodes a and b
ρ	Multilayer perceptron for DDI prediction
ℓ	Denotes the set of all loss functions used in the model
z	Similarity scores of positive and negative samples
τ	Temperature parameter for contrastive learning
M	Number of positive samples for contrastive learning
N	Number of negative samples for contrastive learning

Supplementary Table 2: Hyperparameters and their settings in MKGFlow-DDI.

Hyperparameter	Explanation	Value
Embedding dimension	Node embedding dimension	32
Drug-flow subgraph size	Maximum path length in subgraph	4
Combined graph network layer count	Layers for composite network	2
Subgraph network layer count	Layers for subgraph refinement	2
Learning rate	Optimization learning rate	0.005
Weight decay	L2 regularization coefficient	0.0001
Epochs	Maximum training iterations	80
Batch size	Number of samples per batch	256
Dropout rate	Rate for dropout regularization	0.1
Mask rate for node features	Percentage of masked node features	20%
Gaussian noise std	Standard deviation for Gaussian noise	[0.05, 0.1]
Edge weight perturbation	Relative perturbation for edge weights	± [10%, 30%]

Supplementary Table 3: Experimental results and resource consumption.

Hyperparameter	Explanation	Value
Average subgraph size (DrugBank)	Avg. nodes/edges per drug pair	24 nodes, 32 edges
Average subgraph size (TwoSides)	Avg. nodes/edges per drug pair	28 nodes, 44 edges
Training time (80 epochs)	Time on NVIDIA RTX 3090 (batch=256)	3 hours
Inference time	Per drug pair prediction	15 ms
GPU memory	Peak usage during training	16 GB
Batching strategy	Optimization method	Dynamic subgraph batching with PyG
